# Altruistic responses to the most vulnerable involve sensorimotor processes

**DOI:** 10.3389/fpsyt.2023.1140986

**Published:** 2023-03-10

**Authors:** Brian D. Vickers, Rachael D. Seidler, R. Brent Stansfield, Daniel H. Weissman, Stephanie D. Preston

**Affiliations:** ^1^Department of Psychology, University of Michigan, Ann Arbor, MI, United States; ^2^University of Michigan School of Kinesiology, Ann Arbor, MI, United States; ^3^Department of Medical Education, University of Michigan, Ann Arbor, MI, United States

**Keywords:** empathy, altruism, donation, giving, motor, caregiving

## Abstract

**Introduction:**

Why do people help strangers? Prior research suggests that empathy motivates bystanders to respond to victims in distress. However, this work has revealed relatively little about the role of the motor system in human altruism, even though altruism is thought to have originated as an active, physical response to close others in immediate need. We therefore investigated whether a motor preparatory response contributes to costly helping.

**Methods:**

To accomplish this objective, we contrasted three charity conditions that were more versus less likely to elicit an active motor response, based on the Altruistic Response Model. These conditions described charities that (1) aided neonates versus adults, (2) aided victims requiring immediate versus preparatory support, and (3) provided heroic versus nurturant aid. We hypothesized that observing neonates in immediate need would elicit stronger brain activation in motor-preparatory regions.

**Results:**

Consistent with an evolutionary, caregiving-based theory of altruism, participants donated the most to charities that provided neonates with immediate, nurturant aid. Critically, this three-way donation interaction was associated with increased BOLD signal and gray matter volume in motor-preparatory regions, which we identified in an independent motor retrieval task.

**Discussion:**

These findings advance the field of altruism by shifting the spotlight from passive emotional states toward action processes that evolved to protect the most vulnerable members of our group.

## Introduction

Why do people help perfect strangers? Costly, altruistic aid is thought to have evolved because it improves the fitness of related individuals (1) and the favor is often returned (2). Such aid is thought to be psychologically motivated by feelings of empathy or sympathy, which observers often feel for distressed victims (3–6). These ultimate and proximate mechanisms can be combined if empathy for distressed victims evolved to adaptively promote the care of helpless, related offspring, which was extended during primate evolution to in-group members [e.g., (7–11)]. Our general sensitivity to infant cues has been demonstrated in many ways, through a bias to attend to, find attractive, and help babies and young children (hereafter, neonates) and neotenous looking adults, in comparison to more mature individuals (10, 12–14). The empathic resonance between a distressed individual and observing conspecific has also been demonstrated in many species that care for altricial young [reviewed in (4, 6, 15)].

The perception-action mechanism of empathy relies upon a neural design in which experiencing an affective or physical state activates overlapping brain areas when one observes that state in another [e.g., “empathic pain,” reviewed in (16–20)]. Supporting a link between this neural signature of empathy and altruism, empathic pain is also linked to altruism in multiple fMRI studies (21–25). Further evidence for this mechanism, empathic pain can be blocked by pain analgesia or placebo analgesia [e.g., (26, 27)]. Moreover, when participants felt and observed shocks, they worked harder to reduce shocks to their partner when they responded faster and felt more unpleasant about it, which was reduced by analgesia (28).

These aforementioned studies are important for demonstrating the proximate mechanisms of empathy, but they cannot test when and why people might physically respond to someone in urgent need, which characterizes both offspring care and heroism. Sometimes participants are confronted with confederates in pain and can take over the pain for them [e.g., (29)] or inquire as to their welfare or offer help (30). Regardless, the aid in studies almost never involves an actual physical rescue—a form of altruism that should have been common and important in early instance of altruism toward offspring and in-group members. Additional research is needed to examine whether and how motor responses can be primed by others’ need. According to the Altruistic Response Model, neurohormonal caregiving systems prime motor responses to retrieve victims in situations that resemble offspring need, e.g., when bonded, neotenous, or otherwise helpless victims are distressed and require immediate aid that the observer can provide [reviewed in (10, 31)].

### Existing research on the role of the motor system in human altruism

Some prior research has examined the contribution of motor regions in the brain to empathy or altruism. The motor system is involved in the mirror system representation of others’ actions and states, which supports empathy (32, 33). Motor-related areas are sometimes active in during empathic pain [e.g., middle/dorsal cingulate, premotor (PM) and motor cortex (M1), supplementary (SMA) and somatosensory (S1) cortices, and cerebellum; (22, 25, 34–36)]. Dictator game offers to other participants are correlated with empathic pain responses in cerebellum, S1, and superior parietal lobule (36). Participants who make more deontological moral decisions that avoid harm to others exhibit more PM activation when observing pain to another’s hand (25). Using the same stimuli, observers of pain to another’s hand also show reduced motor-evoked potentials from TMS on their own hand (37)—effects that are modulated, like other empathic outcomes, by trait empathy, in-group versus out-group status, race, culture, and empathy disorders [reviewed in (38)]. When people observe pain in another’s hand or facial expression, they donate more to reduce the confederate’s pain to the degree that the somatosensory area was activated, demonstrated with electroencephalography and impeded by transcranial magnetic stimulation (TMS) and high-definition transcranial direct current stimulation (HD-tDCS) (39). When observing a confederate in pain, participants who offer help respond significantly faster (28). When participants can press a button during empathic pain or control clips for no ostensive reason, they press harder in the pain condition, which reduces the neural empathic pain response (40). These studies demonstrate that motor-related neural regions are activated when people observe another’s action or expression or pain; however, the activation in most cases is interpreted as representing or mirroring the victim’s action or state [or reducing the observer’s personal distress; (40)], rather than preparing the observer to respond to help.

The neural substrates of empathic pain—particularly in anterior midcingulate cortex (aMCC)— are sometimes theorized to represent a preparatory motor response, such as promoting a quick response when a conflict between important desired versus actual states is detected (22, 41). Consistent with this view, there is overlapping activation for felt pain and motor control in aMCC and SMA, supporting their functional overlap in regions that are sometimes active in empathic pain (42). In one study, participants responded faster and activation was stronger in dorsal anterior cingulate cortex (dACC), midcingulate cortex (MCC), and posterior cingulate cortex (PCC) when participants observed a painful implement hitting someone’s hand (but not an innocuous object or when the object missed) (43). However, most researchers assume that aMCC contributes to empathic pain by representing pain, salience, negative affect, or the detection of a problem, without implicating a motor response *per se*, for self or other [e.g., see reviews in (35, 44, 45)]. Some suggest that motor preparation is involved in these situations, but to react quickly as if the victim (43) or to feel better as a contagiously distressed observer (40), not to help the victim. Preparation for action has been implicated in observers’ responses to fearful faces, with increased motor evoked potentials in observers after TMS to primary motor cortex (M1) (46), but this was thought to reflect a fight-or-flight response that protects the observer not one that helps the fearful victim [although the fear expression has been suggested to engender aid due to its neotenous quality; (9)]. Perhaps implicating the need to respond to offspring, observers perform better and exert more pressure on a modified, motoric Wack-a-Mole game when primed with infant cries [compared to adult cries or bird sounds; (14)], demonstrating motor priming from infant distress, but without offering the opportunity to help. Thus, the motor system is often implicated in studies of others’ pain or distress, but without any link in methods or theory to a response on behalf of the victim—a direct, ecological form of aid that should be important and common for survival.

A more direct test of the Altruistic Response Model is seen in bystander apathy paradigms, in which the probability of helping decreases with increasing numbers of bystanders (30, 47). For example, as bystanders increase, observers watching a video of a woman falling exhibit reduced corticospinal excitability (a physiological measure of action preparation) (48) and reduced neural motor system activity in pre- and post-central gyri (49). Moreover, participants who tap the fastest in an unrelated task while watching the same video without bystanders report greater empathic concern and personal distress in daily life (48). Thus, the motor system may be primed by another’s physical need and inhibited during failures to respond. We need further tests of the hypothesis that the altruistic response is promoted by motor-motivational responses to others’ salient need.

In summary, a large body of research shows that observers share in victims’ affective states, and that this neural resonance often promotes altruism and involves motor-motivational processes. But we lack direct tests of the possibility that observing another’s distress and immediate need directly primes costly aid through participation of the motor system. The present study therefore aimed to test the central hypothesis of the Altruistic Response Model, that perceiving a neotenous or otherwise vulnerable victim in immediate need can prime motor-motivational neural activity, which could support a physical response and promote costly giving.

### Overview of the current study

The goal of the present fMRI study was to examine the role of the motor system in costly, altruistic giving. Many caregiving models of altruism suggest that neotenous victims like babies and young children evolved to be the strongest drivers of an altruistic response (10, 12, 50–52). The motor system should be primed to respond with aid when the need is physical, required immediately, and involves a clear action that the observer can make (10, 53). Therefore, we predicted that motor-preparatory responses would be greatest when observing young, vulnerable victims in distress who need immediate, active aid.

To test this hypothesis, we presented participants with charity descriptions that varied along three dimensions. First, they varied in whether they helped neonates or adults (since our hypothesis predicts that people are more motivated to help neonates). Second, they varied in whether they involved immediate aid in the moment or preparatory support from a distance (since our hypothesis predicts that active aid increases with immediate need). Third, they varied in whether they provided heroic or nurturant aid. Both heroic and nurturant aid were expected to be motivating, and to share neural correlates in non-human animals (54), but heroism was expected to engage motor-preparatory regions more because of its naturally physical nature (55). The distinction between heroic and nurturant aid is particularly relevant from a neurobiological standpoint because it is analogous to the documented distinction in caregiving mammals between active (e.g., retrieval) versus passive (e.g., nursing, huddling) offspring care [reviews of the rodent model in (56, 57); applied to human aid in (31)]. After reading about each cause, participants could choose how much money to donate, from the amount earned on that trial.

We expected motor-motivational brain activity to be engaged by our simple charity descriptions even though there were no videos or situations unfolding in real-time because of known similarities between imagining and performing actions (58–60). Our main predictions were that people would donate more to charities that assist neonates with immediate aid, associated with increased BOLD signal during task performance and individual differences in brain gray matter volume in regions that support motor-motivational processes [e.g., dACC and premotor (PM) and primary motor (M1) cortical regions]. To investigate these predictions, we employed an individual differences approach that used each participant’s behavioral donation effect to predict BOLD signal during task performance and gray matter volume. The strength of this behavioral donation effect was also expected to predict BOLD signal and gray matter volume in brain regions activated when participants imagined pulling an object toward the body, akin to the movements required to rescue someone back from danger, through an independent, functional localizer task. Critically, this pattern of results would indicate that altruism involves not only a resonant feeling but also an active, preparatory motor component, at least in situations like a neonate in immediate need.

## Materials and methods

### Participants

Thirty-one (*n* = 15 females; age *m* = 21.62 years, *SD* = 1.48 years) healthy adults completed the fMRI study for $40, plus any money earned during the behavioral donation task that they did not donate. We screened participants *via* the telephone for MRI contraindications. We also ensured that they were 18–30 years old, right-handed, without a history of neurological or psychiatric illness. We excluded one participant due to a power outage at the fMRI center that prevented data collection. We excluded the data from another participant due to excessive head movement during the scan (greater than ± 3 mm). This left 29 fMRI participants for group analysis (15 females, mean age *M* = 21.62 years, *SD* = 2.68). Our sample size was determined from the sample sizes used in similar successful studies of charitable donation [e.g., 19 in (61); 20 in (62); 28 in (63)]; we aimed for the higher end of the distribution of these studies in case participants had to be removed (e.g., for artifacts or poor performance).

The Altruistic Response Model predicts that both donation and motor-motivational neural responses would be largest in response to charities that assist neonates requiring immediate assistance (represented by an interaction effect); moreover, we also manipulated whether such charities provided nurturant or heroic aid. Thus, it was predicted that a two-way interaction would occur, but also possible that a three-way interaction could supersede this. Our fMRI study may not have been powered to detect a three-way behavioral donation interaction, even if people in the scanner did experience the same phenomenon, which could be represented by significant changes in brain activity and gray matter volume. To increase the statistical power to detect a three-way interaction in the behavioral donation data, we also tested 117 introductory psychology subject pool participants (*n* = 39 females, age *M* = 18.97 years, *SD* = 1.33), compensated with course credit and behavioral task earnings that they did not donate. Laboratory participants performed the same task as fMRI participants, with minor modifications for the different context (i.e., they responded on a keyboard instead of with a glove, seated at a computer rather than lying in the scanner, reading each charity only once rather than in repeated trials; they also completed additional surveys). All participants provided informed written consent, and all procedures were approved by the University of Michigan Medical Institutional Review Board.

### Behavioral procedures

#### Charitable donation task

**FIGURE 1:**
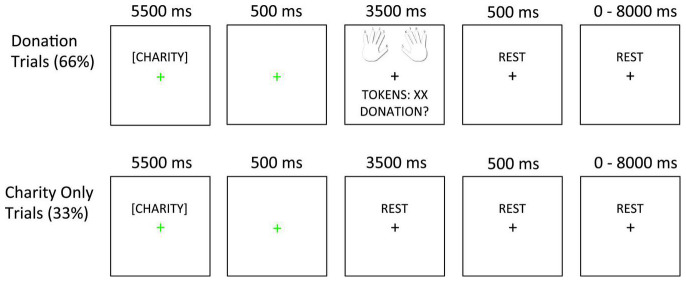
Task design. In Donation trials (66%), the charity description was followed by a 500 ms fixation slide. Next, the decision slide appeared (showing finger-response mappings, tokens earned, and the donation option). In Charity Only trials (33%), the charity description and 500 ms fixation slides were followed by an additional rest period whose duration matched that of the decision slide (3,500 ms). The fixation cursor always changed to green to indicate the onset of the charity description and then back to black after the first fixation slide.

The task is depicted in Figure 1. On each trial, participants read a charity description and earned tokens. Afterward, on Donation trials (66%), participants decided how many of their earned tokens to donate to the current charity. On Charity Only trials (33%), participants only viewed the charity description without donating afterward. In this case, the next screen only included a fixation cross. Including both Donation and Charity Only trials allowed us to dissociate BOLD activity related to viewing the charity description from BOLD activity related to making the donation (64, 65).

There were 12 distinct charitable causes that had been previously piloted in laboratory versions of this study (unpublished data). Each charity was modified into four versions in a 2 × 2 factorial design. In this design, we manipulated whether (1) the aid involved an immediate response or preparatory support for that same helping response and (2) whether the form of aid was heroic or nurturant. In addition, some charities described neonatal victims (babies, children) while others described adults (included as a dichotomous variable during analysis). As an example of our 2 × 2 factorial design, a single charitable cause that rescued adults from the water after their boat capsized was framed in four ways to describe (1) jumping into the water to prevent boaters from drowning (immediate response-heroic), (2) administering warming treatments to boaters pulled from the water (immediate response-nurturant), (3) making harnesses to pull boaters from the water (preparatory support-heroic), and (4) buying warming blankets to care for boaters rescued from the water (preparatory support-nurturant).

Presenting 12 charitable causes in each of four “frames” produced 48 unique stimuli (Supplementary Table 1). Each stimulus was shown twice for a total of 96 trials. These 96 trials were divided across four 24-trial runs, each of which consisted of 16 Donation and 8 Charity Only trials. The four trial types in our experimental design appeared equally often in Donation and Charity only trials and the order of the four trial types was pseudorandomized (using a first-order counterbalancing script in MATLAB^®^) to ensure that each trial type followed the other three with an equal frequency. Inter-trial intervals (ITIs) were jittered between 0 and 8,000 ms (mean = 1,750 ms, sampled from an exponential distribution).

To increase participant motivation and to provide a rationale for earning tokens on each trial, participants were told that their earned tokens were based on how much they attended to each charity description, which we would determine from their neural activity on each trial. In reality, attention was not monitored, and 5–9 tokens were pulled randomly for each donation trial from a uniform distribution. The finger mappings corresponding to donations of 0–9 tokens were displayed on Donation trials with 0 on the left pinky, 1 on the left ring finger, up to 9 on the right pinky. Participants’ donation decision per trial was shown briefly on the screen before that trial’s rest period. Participants knew in advance that they could not see their accumulated earnings or donate more than they earned per trial and that the tokens did not carry over across trials; they also knew that they would receive cash for any undonated tokens after the study along with their standard payment. Participants were led to believe that these were real charities and that their donations would be sent to the relevant charities; in reality, all donated funds were given to a single charity after the study.

#### Charity ratings and personality questionnaires

After the session, participants rated each charity description on several attributes that may predict responses, from 1 (lowest) to 7 (highest). As a manipulation check they rated (1) the level of physicality or bodily energy required for the aid (to indicate what we hereafter call the preparatory support-immediate response) and, (2) how nurturant to heroic the aid seemed (hereafter nurturant-heroic). Other possible attributes that could have been embedded in the descriptions that could have impacted responses were also measured, including how much emotion it evoked, the degree to which it induced the participant to take the helper’s perspective, importance to society and to the participant, and the level of danger involved.

Finally, participants completed several individual difference scales. The Penner Prosocial Battery (PPB) (66) consists of subscales for other-oriented empathy, helpfulness, social responsibility, empathic concern, perspective taking, personal distress, other-oriented moral reasoning, mutual concerns moral reasoning, and self-reported altruism. Three additional scales measured preferences for intuitive/active versus deliberative/rational processing: the Locomotion Assessment Scale (67), the Preference for Intuition and Deliberation Scale (68), and the Need for Cognition Inventory (69). Participants from the laboratory study also completed a behavioral inhibition and activation subscale (70). Only helpfulness, empathic concern, and self-reported altruism from the PPB are discussed further, as all remaining scales were unrelated to our key behavioral donation interaction.

### fMRI procedure

#### Overview

We adopted the following procedure on the day of participants’ fMRI scan. Before going into the scanner, participants performed at least 6 trials of the main donation task and two blocks of an imagined motor retrieval localizer task (described below) on a laboratory computer for practice. Next, participants were positioned in the scanner with a five-button custom response device under each hand, permitting 10 possible donation responses (0 to 9). A T1-weighted scan was acquired along with four runs of the main charitable donation task, one run of the retrieval visualization localizer, and a high resolution SPGR anatomical scan. The total scan duration was approximately 45 min. Afterward, participants rated the charities and completed personality questionnaires for a combined total of 2 h.

#### Data acquisition

Participants viewed the stimuli we created using E-Prime 2.0 software through a mirror, which displayed images projected onto a screen behind the magnet’s bore. Imaging data were acquired using a 3.0 T GE Signa scanner with a standard head coil. To measure the blood oxygenation level dependent (BOLD) signal for each participant in the main task, we acquired 684 functional T2* weighted spiral in/out BOLD fMRI volumes divided evenly across four runs [slice thickness = 4 mm, 29 slices, repetition time (TR) = 2,000 ms, echo time (TE) = 1,700 ms, flip angle (FA) = 30°, in plane resolution = 3.44 × 3.44 mm; 150 volumes for a retrieval visualization localizer described below]. Data were not collected until after the first 5 functional volumes of each run to allow for steady state magnetization. Structural images for data presentation and co-registration were acquired in the same slice locations using a T1-weighted fast gradient echo pulse sequence (TE/FA = 30 ms/90 degrees, in plane resolution = 0.859 × 0.859 mm). High-resolution structural images (voxel size 1 × 1 × 1 mm) were collected using a T1-weighted, spoiled 3D GRE acquisition.

#### Retrieval localizer

To isolate brain areas that may reflect an active motoric retrieval response to help someone in immediate need of a rescue, participants also completed a localizer task that involved imagining reaching out to grab an object and bringing it toward themselves. 15 objects (e.g., books, dinnerware, tools) were shown from the International Affective Picture System database (71). At the beginning of each block, a 2-s cue at the top of the screen told participants to “GRAB” or “WATCH” the item. The cue remained on the screen while five images were presented per block for 4 s each. During retrieval blocks, participants were instructed to visualize reaching out, grabbing the object with both hands, and bringing it back toward themselves, as occurs when caregivers retrieve infants and heroes rescue victims. During watch blocks, participants were instructed to watch the images on the screen passively. Each block type was presented three times in alternating order (reach-watch order counterbalanced across participants) with 20 s rest periods between blocks.

### Analysis

#### Donation behavior

A univariate General Linear Model (GLM) predicted the percent of tokens donated using three fixed factors, their interactions, and one random factor. Subjective charity ratings collected after the fMRI scan revealed discrepancies between how a charity description was categorized *a priori* in our 2 × 2 design versus how participants perceived the charity. For example, all charities that involved a neonate appeared nurturant to participants, even when it was written to be framed as heroic. Therefore, to more accurately represent how participants perceived each charity, analysis used the mean continuous rating for each charity of the perceived preparatory-immediate and nurturant-heroic attributes across all participants (used in both laboratory and fMRI analysis), rather than our *a priori* classification. The GLM used two continuous fixed effects to model the degree to which each participant rated each charity as preparatory-immediate and nurturant-heroic (centered on zero for the rating midpoint by subtracting 4 from each value). An additional dichotomous fixed factor coded whether the charity included a neonate or not (−1 for adults and 1 for neonates). Subject identity was included as a random factor [using the Satterthwaite correction, (72)].

#### fMRI preprocessing

Functional data were preprocessed and analyzed using GLM in Statistical Parametric Mapping 8. The four runs of the charitable donations task were slice time corrected using sinc-interpolation to correct for asynchronous slice acquisition, and spatially realigned to compensate for head movements. We corrected for head motion using the SPM package standard implementations, spm_realign and spm_reslice. The resulting files of the x. y, z, yaw, pitch, and roll realignments per volume were also entered into the statistical analyses as nuisance covariates. The SPGR scan was coregistered to the T1 overlay, and the mean of the functional scans was coregistered to the T1 overlay, with all other functional images coregistered using the same transformation. The SPGR was then spatially normalized to MNI space. Next, we applied the parameters generated to normalize the SPGR to normalize the functional images. Finally, the functional images were spatially smoothed using a Gaussian filter of 5.0-mm full width half maximum (FWHM).

#### fMRI analysis of retrieval localizer

The retrieval localizer was analyzed in a block-wise fashion. Regressors were included for watching, retrieving, and rest blocks (with the duration equal to the time they were on the screen, 20 s each), along with six nuisance regressors for rotation and translation head movement parameters. Regressors were convolved to both the canonical HRF as well as the temporal derivative. The contrast of interest compared retrieving to watching blocks using a threshold of *p* < 0.05 with FWE correction. We used this contrast to create regions of interest (ROIs) for subsequent orthogonal analyses.

#### fMRI analysis during the charitable donation task

At the first level of analysis, we modeled the Charity Viewing and Donation periods separately in the GLM. Each trial type was modeled with duration equal to the time displayed (Charity Viewing = 5.5 s, Donation = 3.5 s). We also included the temporal derivative for each trial type to decrease model error variance. Finally, we included nuisance regressors for the intercept of each run (4 regressors) and for the x, y, z translation and yaw, pitch, roll rotation head movement parameters generated during realignment (6 regressors per run).

The primary result from the behavioral data was a three-way interaction between the response being preparatory-immediate, nurturant-heroic, and whether the victim was a neonate or adult. Thus, fMRI analysis also focused on the neural correlates of this guiding three-way interaction. To this end, we calculated a single behavioral parameter for each participant, representing the strength of their own three-way interaction based upon their behavioral donation data. We then conducted two one-way, random effects analyses of covariance (ANCOVAs). Both ANCOVAs had the three-way interaction term from the behavioral donation data as the continuous covariate at the second level. The first ANCOVA used the first level model results from the charity-viewing period and the corresponding temporal derivative for all participants. The second ANCOVA used the first-level model results from the donation period and the corresponding temporal derivative for all participants. Thus, these analyses revealed brain regions in which activity during Charity Viewing (ANCOVA 1) and Donation (ANCOVA 2) periods varied with the strength of the behavioral three-way interaction across participants.

Additional models including the main effects of charity viewing or donation periods contrasted against a baseline and other simple effects are reported for transparency in the supplement, with results in Supplementary Tables 2, 3.

#### Region of interest (ROI) analyses

Two ROIs were taken from the retrieval localizer, centered on two significant peaks that passed the FWE *p* < 0.05 threshold for retrieving > watching: left inferior parietal lobule (IPL) (−42, −44, 50) and left premotor area (PM; −24, −8, 56). Five additional ROIs were identified from a prior study of altruistic charity donations, which compared donations to monetary rewards, hereafter referred to as the *a priori* ROIs [from Supplementary Table 3 in (61)]. These ROIs included subgenual cortex (−2, 14, −5), ACC (−1, 19, 42), ventral striatum/septal region (VS; −2, 5, −2), and right (63, −31, 14) and left (−48, −53, 6) superior temporal sulcus (STS). Threshold *p*-values related to the ROI analyses were Bonferroni corrected by the number of ROIs [as in (73)]. This yielded a voxel threshold of *p* < 0.0071 and we adopted a minimum cluster size threshold of 10 voxels. All ROIs were 13.0 mm radius spheres centered on the significant peak coordinate.

#### Exploratory whole-brain analyses

To explore neural correlates of the donation interaction outside of the ROIs, we also performed whole brain analyses for the three-way interaction. These analyses were voxel-level thresholded at *p* < 0.0001 uncorrected with a minimum cluster size of 10 voxels. This threshold is less stringent than a corrected *p*-value but appropriate for a purely exploratory test of a three-way interaction in the brain.

#### Standard voxel-based morphometry (VBM) protocol

To explore possible morphological differences across participants associated with the three-way behavioral donation interaction, we conducted a VBM analysis (74). Each participant’s SPGR scan was segmented into gray matter, white matter, CSF, and other non-brain partitions and warped to MNI space. Images were then smoothed using a 10 mm FWHM Gaussian kernel. VBM ROI analyses employed the same ROIs and thresholding as in the functional ROI analysis (i.e., Bonferroni correcting by the seven ROIs for a voxel level threshold of *p* < 0.0071; minimum cluster size threshold of 10 voxels). As with the functional analysis, we also explored potential areas of relevance outside of the *a priori* ROI in the whole brain with the same thresholding as the functional whole brain analysis (GLM, with total intracranial volume as a between-participants covariate to control for size differences in intracranial space; threshold: *p* < 0.001 uncorrected; cluster size: 10 voxels).

## Results

### Task and behavioral donations

**FIGURE 2:**
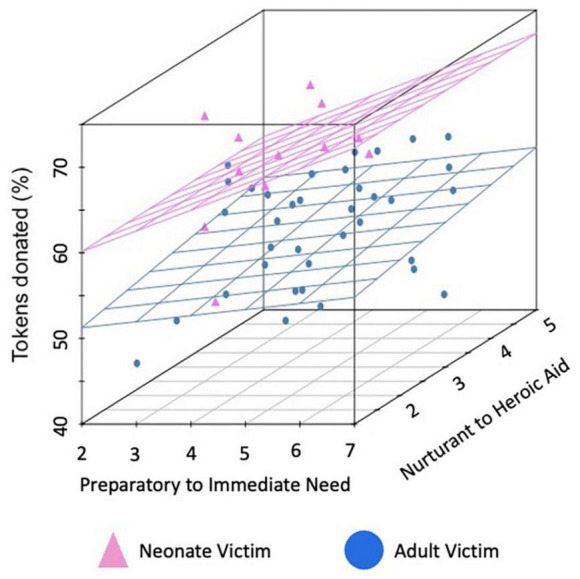
Mean donations per charity by condition. Mean percent of tokens donated for each of the 48 charities. Charities that assisted neonates are represented in pink while those assisting adults are in blue. Data are plotted on the first axis by the degree participants (on average), rated the charity as sounding more like preparatory support provided at a distance (lower ratings) versus an immediate response in the moment (higher ratings) and on the second axis by the degree that it sounded more like nurturant (lower ratings) versus heroic (higher ratings) aid (both rated from 1 to 7).

**FIGURE 3:**
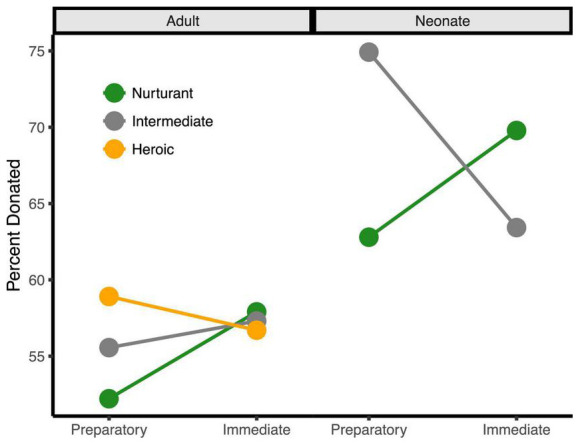
The three-way behavioral donation effect. Estimated mean percent of tokens donated by condition (percentages are used since token earnings varied by trial). Charities that assisted adults are on the left half while those that assisted neonates are on the right. For visualization only, charities < 1.5 *SD*s from the neutral midpoint were classified as Nurturant (green), charities > 1.5 *SD*s from the midpoint were classified as Heroic (orange), and charities at the midpoint were classified as Intermediate (gray). Charities assisting neonates were always rated as more nurturant and not highly heroic, explaining the absence of the orange line on the right side. This bias likely results from the fact that infants and children are so highly associated with nurturance that it was difficult to dissociate neonates from nurturance.

Participants’ costly monetary donations to the charities supported the three main hypotheses from the Altruistic Response Model (see Figures 2, 3). Participants donated significantly more to charities that assisted neonates as compared to adults (69.2% of tokens in neonate charity trials versus 56.4% of tokens in adult charity trials), *b* = 12.74, *se* = 1.72, *t*(7329) = 7.41, *p* < 0.0001, 95% CI = [9.37, 16.11]. Further, participants donated more to the extent that they perceived the charity as providing an immediate response at the time of need in contrast to a preparatory response provided at a distance, *b* = 0.57, *se* = 0.29, *t*(7329) = 2.04, *p* = 0.047, 95% CI = [0.008, 1.139]. Finally, participants donated more to the extent that they perceived the charity as providing heroic as compared to nurturant aid, *b* = 1.19, *se* = 0.48, *t*(7329) = 2.47, *p* = 0.014, 95% CI = [0.25, 2.13]. These three main effects support the idea that humans, as a caregiving mammal, evolved to be motivated to act when a helpless neonate requires immediate, physical aid.

Importantly, these three main effects were qualified by a three-way interaction, *b* = −4.14, *se* = 1.47, *t*(7329) = −2.83, *p* = 0.005, 95% CI = [−7.02, −1.28]. This interaction occurred because the degree that participants donated more to charities for neonates over adults only increased with the immediacy of the response when the aid sounded nurturant, *b* = −4.36, *se* = 1.86, *t*(7329) = −2.34, *p* = 0.019, 95% CI = [−8.01, −0.71] but immediacy did not increase the response when the aid sounded heroic, *b* = 1.31, *se* = 1.24, *t*(7329) = 1.05, *p* = 0.292, 95% CI = [−1.12, 3.74]. This occurred because charities that assisted neonates were always perceived as more nurturant and were not perceived as heroic, whereas heroic charities already sounded immediate and, thus, could not further benefit from this additional immediacy framing. These behavioral effects remained significant after controlling for participant sex and response time (all *ps* < 0.05).

Because this three-way interaction was the primary behavioral donation result, which qualifies the lower-level main effects and interactions, subsequent analyses focused on this effect. We extracted the behavioral donation interaction effect term for each participant and correlated it with charity ratings, trait data, neural activity, and structural morphometry. The three-way behavioral donation interaction term per participant, therefore, served as an individual differences variable that was largest when participant donations increased for neonates with the immediacy of the response, particularly for nurturant forms of aid.

### Behavioral donations correlated with charity ratings

To understand the nature of the three-way behavioral donation interaction, we first examined whether its strength varied with participants’ ratings of other charity characteristics. The strength of the behavioral donation interaction increased when the charity elicited more emotion, placed participants into the perspective of the hero, and sounded more important to society and to participants personally. Each of these variables entered individually as a covariate in the GLM eliminated the three-way behavioral donation interaction, *bs* < | 1.00|, *ts*(7328) < | 0.70|, *ps* > 0.450; each also predicted donation amounts directly and correlated with the other three ratings [all *rs* > 0.45, *ts*(107) > 5.00, *ps* < 0.001]. Only the degree of perceived danger for the helper was statistically unrelated to the three-way behavioral donation interaction, *b* = −2.08, *se* = 0.74, *t*(7328) = −2.83, *p* = 0.005. In sum, because emotion, taking the hero’s perspective, and perceived societal and personal importance each predicted giving and were intercorrelated, these attributes appear to be mutually reinforcing and to co-occur when people are inspired to help.

### Behavioral donations correlated with personality traits in females

None of the prosocial traits significantly predicted the three-way behavioral donation interaction [PPB empathic concern was marginal, *b* = 0.009, *se* = 0.005, *t*(113) = 1.73, *p* = 0.087; all others, *ps* > 0.130]. However, females with higher behavioral donation interaction scores reported being more helpful in daily life, *b* = 0.012, *se* = 0.005, *t*(110) = 2.31, *p* = 0.023, and marginally more altruistic, *b* = 0.012, *se* = 0.007, *t*(110) = 1.82, *p* = 0.072 than males [males: *b* < −0.003, *se* > 0.003, *t*(110) < −0.73, *p* > 0.340; gender interactions: *ts* > 2.00, *ps* < 0.05]. No other traits or effects of gender interacted with the three-way behavioral donation interaction (all *ts* < 1.00, *p* > 0.300).

### Behavioral donations correlated with neural measures

**FIGURE 4:**
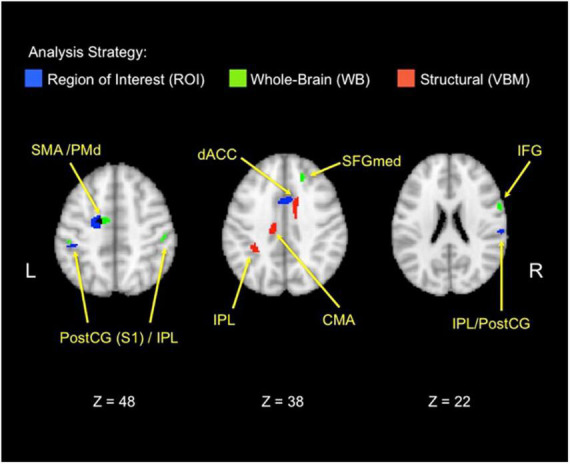
Functional and structural correlates of the behavioral donation interaction. The figure displays brain areas that significantly increase activation with the strength of the three-way behavioral donation interaction (higher for neonates as immediacy increases for nurturant aid but not heroic). Results are combined across analysis strategies including ROI analyses from the retrieval localizer and the *a priori* regions from a prior study (blue), whole-brain functional regions (green), and voxel-based morphometry (VBM) (red) analyses. The VBM threshold was dropped for the figure only to facilitate viewing to 0.005, uncorrected. CMA, cingulate motor area; PostCG(S1), postcentral gyrus/primary somatosensory area; IFG, inferior frontal gyrus; IPL, inferior parietal lobule; PMd, dorsal premotor area; SFGmed, medial superior frontal gyrus; SMA, supplementary motor area.

Table 1 and Figure 4 depict the neural regions associated with the three-way behavioral donation interaction using the ROIs and whole-brain analyses of functional and structural data. Notice that all but two of the results reflect activation during Charity Viewing, wherein participants simply read about the charities before donating *via* a motor response, which reduces the likelihood of a response confound in the data.

**TABLE 1:** Brain areas associated with the three-way behavioral donation interaction.

Period	Gyral label	Functional subarea	Voxels	*t*	*x*	*y*	*z*	Analysis source
Charity viewing	IFG	IFG	67	4.99	62	6	22	WB
	SFGmed		34	4.52	18	34	40	WB
	dACC/MFG	SMA	85	4.97	−12	−6	48	WB*
		dACC	90	3.54	−4	8	42	ROI-A
		dACC	-	3.22	8	10	40	ROI-A
	preCG/MFG	SMA/PMd	280	4.88	−14	−6	48	ROI-L*
		M1	-	3.50	−32	−14	62	ROI-L
		SMA	-	3.45	−12	−8	60	ROI-L
	SFG	M1	-	2.65	−22	−6	64	ROI-L
	postCG/IPL	S1	82	4.99	52	−26	54	WB
		IPL	26	4.34	−46	−30	42	WB
		S1	-	4.03	−50	−28	50	WB
		IPL	59	3.82	−44	−32	46	ROI-L
		IPL	-	3.04	−48	−36	42	ROI-L
	postCG/IPL	S1	40	3.25	62	−24	24	ROI-A
		IPL	-	2.73	56	−22	20	ROI-A
	IPL	IPL	-	3.21	66	−28	26	ROI-A
Donation	IOG	OFA	41	−4.96	40	−80	−12	WB
	IPL	IPL	14	−2.72	−42	−54	52	ROI-L
Structural VBM	dACC/MFG	dACC	15	3.57	15	12	40	WB
		CMA	34	3.89	−14	−19	37	WB
	IPL	IPL	38	3.38	−33	−42	40	ROI-L

Brain areas associated with the three-way behavioral donation interaction (higher for neonates when aid is immediate if nurturant). Results are displayed from three analyses (in the last column, labeled analysis source): ROIs from the functional retrieval localizer task (ROI-L) and *a priori* regions from an existing charitable donation study (ROI-A), whole-brain (WB), and VBM. Positive *t* values represent areas that increase with the donation interaction; negative *t* values represent the reverse. Positive *x* values are in the right hemisphere, negative in the left. SMA/PMd emerged in both WB and ROI-L analysis, marked with an asterisk in the last column. CMA, cingulate motor area; dACC, dorsal anterior cingulate cortex; IFG, inferior frontal gyrus; IPL, inferior parietal lobule; IOG, inferior occipital gyrus; M1, primary motor area; MFG, middle frontal gyrus; OFC, orbitofrontal cortex; PMd, dorsal premotor area; postCG, postcentral gyrus; preCG, precentral gyrus; S1, primary somatosensory cortex; SFG, superior frontal gyrus; SFGmed, medial superior frontal gyrus; SMA, supplementary motor area.

#### Regions of interest (ROI-L) from the retrieval localizer

In line with the Altruistic Response Model, the three-way behavioral donation interaction scaled with activity in the ROIs from our motor retrieval localizer, wherein participants imagined reaching out to pull an object back toward themselves as one would do during a physical rescue (Table 1, labeled ROI-L). The strength of participants’ three-way behavioral donation interaction increased during Charity Viewing with neural activity in PM (the dorsal portion, PMd), M1, SMA, and inferior parietal lobule (IPL) and decreased during the Donation period in the IPL from the ROI-L, based upon the independent motor retrieval localizer. In addition, VBM analysis revealed that gray matter volume in the IPL cluster from the ROI-L retrieval localizer increased with participants’ three- way behavioral donation interaction.

#### *A priori* regions of interest (ROI-A) from the literature

Analysis using *a priori* ROIs from a prior study of charitable giving (61) support the hypothesis that the three-way behavioral donation interaction is associated with motor-motivational processes as activation increased during Charity Viewing in dACC, S1, and IPL (Table 1, labeled ROI-A).

#### Exploratory whole-brain (WB) analyses

Exploratory whole-brain (WB) analyses revealed three additional findings. The strength of the three-way behavioral donation interaction increased during Charity Viewing in inferior frontal gyrus (IFG), decreased during the Donation period in occipital face area (OFA), and increased with gray matter volume in the whole-brain VBM analysis in cingulate motor area (CMA) and dACC (just caudal and lateral to the cluster that emerged in the *a priori* ROI-A analysis) (Table 1, labeled WB).

## Conclusion

The science of human altruism has reached broad consensus that people help when it benefits them or their genes, often mediated by an empathic emotional state that adaptively promoted the survival of helpless offspring and in-group members [e.g., (7–11)]. This ultimate-proximate explanation of altruism has been supported by evidence of the other-oriented motivational state of empathy, with neurophysiological correlates in self-other neural overlap, that promotes altruism. To date, however, there is little research on forms of altruism that were common in early hominid life, such as the direct, physical response to help someone in immediate need.

According to the Altruistic Response Model, as a caregiving mammal, people evolved a powerful motivation to retrieve neonates and similarly vulnerable, distressed individuals needing immediate aid that the observer can provide (10, 31). Many features of this model are shared with other evolutionary theories of empathy and altruism, supported by evidence of our bias to appreciate, approach, and help neonates and evidence of the ways that neurohormones like oxytocin and prolactin promote social bonding [e.g., see (13, 51, 75–77)]. The Altruistic Response Model, based on research in non-human animals, uniquely predicts that need situations that resemble the needs of offspring directly prime the motor system, assuming expertise for the necessary response. To date, research links empathy and altruism to activation of motor-motivational neural regions or facilitated motor action, but usually interpreted as a sign of mirrored action or a self-oriented response, not one that helps the victim.

The present study was designed as an initial test of the hypothesis that observing a neotenous individual in immediate need of assistance—a stimulus that should motivate people to respond physically—will activate the motor system more than other types of altruism, in accordance with a prepared response. Our findings provide novel, initial support for this hypothesis and advance the field by shifting the spotlight away from more abstract and artificial types of aid toward more ecological forms that may have evolved to protect close others in the greatest need.

### The key role for action in decisions to give

Several of our findings support the role of the motor system in the response to the immediate need of neonates. The behavioral donation data support the model’s key prediction that people are more inspired to help when the situation resembles the ancient need to protect and care for helpless, distressed offspring in immediate need. Specifically, study participants donated more when (1) the charity assisted neonates rather than adults, (2) when the aid was delivered as an immediate response in the moment of need rather than as preparatory support, and (3) when the aid sounded heroic as compared to nurturant. Of importance, these three main effects were qualified by a three-way interaction: the degree that people gave more to neonates than adults increased with perceived immediacy when the aid sounded nurturant, whereas it was not impacted by immediacy for heroic aid. This three-way behavioral donation interaction reflected the fact that participants naturally construed assistance to neonates as nurturing and heroism as immediate, because these variables naturally co-occur in daily life.

As predicted, the strength of the key three-way behavioral donation interaction increased with neural activity in brain regions associated with action, performance monitoring, and motor imagery, including the SMA/PMd, dACC, M1, S1, and IPL. Supporting the interpretation that this brain activity is associated with a motor act, which might serve to rescue the victim, several of these regions also emerged from an independent localizer task wherein the same participants imagined reaching out to retrieve an object to pull it back toward them (SMA/PMd, M1, and IPL). The strength of three-way interaction in the behavioral data also correlated positively with gray matter volume in multiple motor-motivational brain regions, including dACC, CMA, and IPL. These findings provide initial support for the idea that people may have evolved empathy or sympathy along with the motor-motivational processes needed to respond in salient conditions.

### Interpreting motor-motivational activation

Our interpretation of the results as reflecting motor-motivational processes is consistent with current theories for the role of motor and posterior medial frontal regions in behavior. The primary motor cortex (M1) is associated with motor control and motor sensation from actions (78). The posterior medial frontal cortex is involved in performance monitoring before, during, and after action is taken (79, 80). Moreover, the regions activated during our retrieval localizer are similar to the neural substrates for motor reaching [e.g., PMd and IPL; (81)]. These considerations further support the view that motor processes participate in responses to need and decisions to give. As we did not contrast our motor reaching localizer task with another type of motor task, we cannot firmly conclude that the motor-related brain activity that correlated with costly giving reflected a “retrieval” response (rather than another action like pushing or escaping). Future research should further specify the nature of this motor-related activity.

Multiple factors suggest that the relationships we observed between the three-way behavioral donation interaction and motor-motivational brain activity did not merely reflect processes required to press buttons for donating. For example, these relationships were observed even when participants only read the charity descriptions, before the donation decision period (which did not occur on all trials). Moreover, activation was relatively evenly distributed between right and left cerebral hemispheres, even though all participants were right-handed and responded with their right hand. Activation was also located in regions that were isolated in a separate localizer task wherein the same participants imagined retrieving objects. Finally, multiple regions associated with the three-way behavioral donation interaction were also larger in gray matter volume, and brain size does not change as a function of task-related responses. These findings argue against the view that our results simply reflect motor execution processes associated with task-related responses.

Prior research suggested that motor-motivational activation to distress or pain reflects the observer “trying on” the actions of the victim, which can increase giving through heightened empathy and perspective-taking [e.g., (35)]. This is probably the case for designs that depict hypothetical victims that are passively observed without the opportunity to respond. But in our study, charities that described adult victims who were more similar to the participants, and who required highly physical rescues, elicited less giving and motor-motivational activation compared to situations involving neonates who were not in motion and required more nurturant aid. Thus, this design may have elicited motor-motivational responses to rescue or protect victims, potentiated by neonates and immediacy. Further work is needed.

### Implications for prior theories of altruism

#### Evolutionary models

Given that people donated more to charities that assisted neonates than adults, our data broadly support models of altruism that involves the fundamental, ancient need to care for helpless neonates [e.g., (7–10)]. The Altruistic Response Model uniquely focuses on the role of motor preparation for action in response to the salient cues of caregiving that require immediacy and expertise (10).

Evolutionary theories of altruism that focus on peer cooperation or reciprocation, or that require complex social-cognitive, mindreading, or cost-benefit processes [e.g., (82–85)], do not explain this form of altruism, which favors neonates and motor-motivational neural responses. For example, our adults in need received significantly less help than the neonates who could not cooperate or reciprocate. Moreover, the associated neural regions that emerged are homologous with those identified in non-human primates to support action mirroring, empathic pain, and motor reaching and grasping [e.g., (81, 86)]—processes that are not specific to humans or cognitively sophisticated. Cost-benefit analyses also do not appear necessary for this type of altruism, since our participants gave less to adults who would die without a rescue than to children needing long-term care [in specific opposition to proscriptions of “effective” or “pure” altruism, wherein help should maximize benefit, see (62)]. Complex cognitive decision processes exist and can support empathy and altruism when the task is different, but they are not required for immediate responses to physical need and did not drive the earliest forms of aid. Moreover, data suggest that sensorimotor activation to others’ pain even predicts individual differences during moral dilemmas and economic games (25, 36)—tasks that people often assume are more cognitive than empathic pain. In summary, our data support comparative evolutionary views that allow for a homology across species, through intuitive but adaptive responses, that are not specific to humans or their cognitive capacities.

#### Self-oriented altruism

Our data are consistent with the “warm glow” theory of altruism, whereby people give because they expect to feel good (62, 87). For example, rescuing a neonate, and the ensuing close contact, is rewarding and reinforces responding (10). Consistent with the warm glow theory, we observed activity in the reward-related ventral striatum (VS) during task performance, as compared to resting baseline periods, and when charities assisted neonates with increasing heroism (Supplementary Tables 2, 3). However, VS activity was not significantly correlated with our key donation interaction, suggesting that warm glow plays a role in altruism generally without being specific to salient, active aid.

A prior study linked motor responses to self-oriented altruism because harder button pressing to others’ pain reduced the neural signature of empathic pain and perhaps selfishly reduced personal distress (40). The Altruistic Response Model instead presumes that action and helping were artificially dissociated in their task whereas normally forceful responses to pain can prepare one for action, to withdraw from your own pain or respond to another’s, depending on the situation.

#### Empathic pain

We replicated findings from social neuroscience that the anterior insula (AI) and pACC/aMCC are involved in empathic pain (18, 20, 35, 88), which is sometimes linked to giving (21–24). These regions were more active during our task compared to resting baseline (Supplementary Table 2). However, as with the VS, this empathic pain activity was not tied to our key donation interaction, in which people gave more to neonates needing nurturant aid with increasing immediacy. Instead, our three-way behavioral donation interaction was associated with midline regions that are more dorsal and caudal to those typical of empathic pain. Sometimes empathic pain studies report motor-motivational activation in regions that overlap with our key effect. However, activity in these regions has been interpreted as representing the victim’s pain or mirroring the victim’s action (34, 35, 43), not as evidence of a prepared, helpful response. Thus, the neural mechanisms underlying active aid appear dissociable from those underlying empathic pain, even if both processes normally co-occur, serially and concurrently, when observing another’s pain.

Several additional findings speak against a pure “empathic pain” interpretation of our findings. First, our stimuli were brief, written phrases that did not focus attention on a limb, location, or sensation in the victim—features that are thought to explain sensorimotor engagement during empathic pain paradigms (35). Second, motor regions associated with our donation interaction were also activated when participants imagined retrieving an object, suggesting that they could represent reaching out to rescue a victim back from danger, rather than sharing their pain. Third, our adult participants donated more to assist neonates than adult victims, even though empathic brain activity usually increases for similar others [e.g., in age, body size, etc., see (89)]. Fourth, the three-way behavioral donation interaction increased with observer reports that they placed themselves into the perspective of the hero, not the victim. Fifth, the movement of the victim in each situation was fixed, whereas the movement of the responder was varied; thus, changes by condition cannot reflect differential amounts of simulated movement, as if the participant is the victim. In sum, we replicated activation of the empathic pain circuit across trials, but motor-motivational activation associated with our key donation interaction may reflect more of a complementary response to rescue someone with salient need. The distinction between mirroring the victim, acting to help one’s self, and acting to address the victim’s need requires further research. Likely motor action is primed and prepared regardless, but the subsequent pattern of downstream activation (e.g., specific effectors, approach/avoidance) changes depending upon the nature of the goal-oriented response (90–93).

#### Charitable giving

We replicated the neural substrates of charitable giving. Prior studies of charitable giving reported an association between donation decisions and mesolimbocortical processes, particularly in the ventromedial prefrontal cortex [VMPFC, (94)], and nucleus accumbens (61, 62, 95, 96). Our whole-brain comparison of task versus resting baseline activated multiple areas associated with decision and reward processes (Supplementary Table 2) in prefrontal cortex, striatum, ACC, insula, thalamus, and hypothalamus. This circuit is also activated by images of neonates (51, 97, 98) and their cries (99, 100)—particularly OFC, amygdala, nucleus accumbens, and cingulate cortex. Thus, our findings are consistent with the view that decisions to give are processed through domain-general, reward-based decision circuits, which are activated by neonates and by altruism. We augment this general view with an additional phenomenon, whereby neonates in immediate need may prime responses that also promote financial giving.

### Future work

The fact that our key three-way behavioral donation interaction existed and was used as an individual difference measure means that the bias toward neonates or for nurturant, immediate care varies across people. This motivation is likely enhanced by factors that also promote early caregiving, such as close contact and stimulation, empathic care, reproductive maturity, and caregiving experience (5, 10, 11, 101). Consistent with this, the relationship between our behavioral donation interaction and self-reported daily altruism was stronger in females than in males, in accordance with expected gender differences in primate theories of caregiving (7). In real-life rescues, motor expertise for the required act is also expected to be essential to a response (10), which we did not test. Future work should examine individual difference and life history factors that enhance responding, expecting stronger responses in females, individuals of reproductive age, caregivers, and those with motor expertise.

Given the intense scrutiny on the role of the cingulate cortex in self-pain and empathic pain [e.g., (44, 45, 102)], it seems worthwhile to investigate the possibility that activation in sensorimotor and pACC/aMCC regions during empathic pain paradigms does not just represent salience, surprise, or motor resonance, but also directed attention to facilitate responses to unexpected, salient events that require immediate action (41, 79, 103). Presumably, this neural capacity evolved to help organisms escape personal injury, which was extended to understand and assist others, but this requires further investigation. The situation should alter downstream responses from quick attentional shifts that generally prime action.

Prior studies found important differences in the empathic pain response by race, nation, and socioeconomic status. For example, in China, charitable donations were predicted by neural empathic pain responses (IFG and secondary somatosensory cortex), but only in high subjective socioeconomic status (SES) participants; in fact, correlations between donations and activation in those regions were negative for low SES participants (22). In the United States, pain images activated left premotor cortex along with insula and ACC; however, the in-group race bias for empathy was stronger for African Americans and associated with medial prefrontal cortex activation; in contrast, the in-group bias of Caucasian participants was more associated with the motor-motivational activity observed here and during empathic pain (in pre and post-central gyri and cerebellum, along with insula and other areas) (23). In England, older adults were more willing than young adults to exert physical force to benefit others (104). Empathy and altruism clearly differ by culture and are biased toward more similar, familiar, and in-group members; as such, this work must be replicated in other groups and contexts. Our results were also associated with realistic but fictional charities, some of which were observed twice by scanner participants [after (61); laboratory participants only viewed them once]. Results could be stronger if participants view real, familiar charities, once each, before having to donate their own money (i.e., over tokens or house money). Given that deontological moral reasoning has been shown to correlate with responses to empathic pain in the past (25), it would be worthwhile in the future to try to map the deontological-utilitarian distinction on to the immediate-preparatory axis, perhaps using a process dissociation procedure [e.g., see (105, 106)].

### Final conclusion

Our findings provide preliminary support for the idea that altruism evolved through a mechanism designed to promote active care and protection for our own helpless infants or those who are similarly vulnerable, distressed, and needing immediate aid that we can provide. In this way, while people often reflect on a more passive tenderness or sympathy for those less fortunate, and mirror their suffering, they may also be physically inspired toward victims under specific, ecological conditions. Even if this instinct sometimes leads to “irrational” or costly decisions to assist a precious few, these are not “poor” decisions because they are critical to survival. Knowledge of this instinct can be applied to promote giving in situations that are urgent but appear to be longer-term and more distant, like climate change or heart disease. Emphasizing the need of attractive and vulnerable individuals in the moment, alongside effective and concrete responses, can help bridge this gap.

## Data availability statement

The raw data supporting the conclusions of this article will be made available by the authors, without undue reservation.

## Ethics statement

The studies involving human participants were reviewed and approved by University of Michigan IRB. The patients/participants provided their written informed consent to participate in this study.

## Author contributions

SP: study concept and manuscript revision. SP, RDS, DW, and BV: design and methods. BV: participant testing and analysis and manuscript draft. RBS: additional analyses. All authors contributed to the additional editing and approved the submitted version.
