# Supplementary material for: Altruistic responses to the most vulnerable involve sensorimotor processes
Source: Front Psychiatry. 2023 Mar 10;14:1140986. doi: 10.3389/fpsyt.2023.1140986 (PMC10036353; doi:10.3389/fpsyt.2023.1140986)
Supplement: Supplementary file 1 [file Table_1.docx]

**Table S1. Complete charity descriptions used in the charitable donation task.**

The first column indicates whether the charity is framed as an immediate response or preparatory support, the second column indicates whether it is framed as heroic or nurturant aid, and the third column indicates whether the charity assists an adult (A) or neonate (N). Charities describing the same cause have the same number across conditions.

| **Condition** | | **Neonate (N) or Adult (A)** | **Charitable Cause** | **Description** |
| --- | --- | --- | --- | --- |
| Immediate Response | Heroic | A | 1 | Locating and pulling trapped people from collapsed buildings with trained canine squads |
|  |  | A | 2 | Jumping in to retrieve people whose boats capsized in a storm with a special harness |
|  |  | A | 3 | Bringing people to emergency water wells during draughts to treat acute dehydration |
|  |  | A | 4 | Emergency removal of biological agents from the bloodstream of soldiers during wartime |
|  |  | A | 5 | Trained firefighters rushing into burning buildings to save people trapped inside |
|  |  | A | 6 | Mobilizing rescue mission teams to free American hostages that are being held abroad |
|  |  | A | 7 | Rappelling helicopter teams that retrieve people buried in snow after avalanches |
|  |  | A | 8 | Emergency teams to remove women from domestic abuse threats to remote safe houses |
|  |  | A | 9 | Rescuing abused cats and dogs from homes that are dangerously unsafe or unsanitary |
|  |  | N | 10 | Helicopter deliveries to bring antibiotics to infected children in remote Central America |
|  |  | N | 11 | Emergency helicopter transportation to bring premature babies to intensive care units |
|  |  | N | 12 | Bringing children whose parents died to temporary homes until new families are found |
|  | Nurturant | A | 1 | On-site bandaging for people with broken bones who were pulled from collapsed buildings |
|  |  | A | 2 | Administering warming treatments to boaters rescued from capsized boats during storms |
|  |  | A | 3 | Giving rehydrating treatments to people suffering acute dehydration during a draught |
|  |  | A | 4 | Providing bedside demethylation treatment to soldiers exposed to biological agents |
|  |  | A | 5 | Treating serious burns as part of a firefighting team on site at burning buildings |
|  |  | A | 6 | Providing post-traumatic therapy sessions for American hostages who were held abroad |
|  |  | A | 7 | Providing hypothermia treatments at rescue stations for people retrieved from avalanches |
|  |  | A | 8 | Caring for and comforting women who are victims of chronic domestic abuse at safe houses |
|  |  | A | 9 | Tending the wounds of abused cats and dogs recovered from dangerous or unsanitary homes |
|  |  | N | 10 | Administering antibiotics to children suffering from infections in remote Central America |
|  |  | N | 11 | Giving touch therapy to treat babies who were born prematurely in intensive care units |
|  |  | N | 12 | Caring for children whose parents died unexpectedly while new families are located |
| Preparatory Support | Heroic | A | 1 | Training canine squads to locate and remove people buried in collapsed buildings |
|  |  | A | 2 | Making special harnesses to safely rescue boaters from capsized boats during storms |
|  |  | A | 3 | Designing advanced well systems that can be used abroad during emergency draughts |
|  |  | A | 4 | Developing techniques to remove biological agents from soldiers' bloodstream during war |
|  |  | A | 5 | Training firefighters to safely search burning buildings for possible trapped inhabitants |
|  |  | A | 6 | Brokering diplomatic agreements to release American hostages who are being held abroad |
|  |  | A | 7 | Training helicopter teams to repel down to retrieve people buried under avalanches |
|  |  | A | 8 | Building remote safe houses to protect at-risk victims of chronic domestic abuse |
|  |  | A | 9 | Building shelters for abused cats and dogs removed from unsafe or unsanitary homes |
|  |  | N | 10 | New procedures to treat infected children in remote Central America without electricity |
|  |  | N | 11 | Developing surgical techniques to save premature babies in the intensive care unit |
|  |  | N | 12 | Building foster homes to house children whose parents died while new families are located |
|  | Nurturant | A | 1 | Training medical staff to treat broken bones of people found in collapsed buildings |
|  |  | A | 2 | Purchasing warming blankets to treat boaters rescued from capsized boats during storms |
|  |  | A | 3 | Making rehydration packets to give to people suffering acute dehydration in draughts |
|  |  | A | 4 | Training nurses to perform bedside demethylation to soldiers exposed to biological agents |
|  |  | A | 5 | Training hospital staff to provide long-term care for people burned in building fires |
|  |  | A | 6 | Training therapists to provide post-traumatic counseling to American hostages held abroad |
|  |  | A | 7 | Preparing hypothermia kits to warm people recovered from avalanches in the backcountry |
|  |  | A | 8 | Managing remote safe houses to keep at-risk victims of chronic domestic abuse safe |
|  |  | A | 9 | Purchasing bandages for abused cats and dogs recovered from unsafe or unsanitary homes |
|  |  | N | 10 | Teaching parents to administer antibiotics for kids' infections in remote Central America |
|  |  | N | 11 | Training mothers how to perform touch therapy for their babies who were born prematurely |
|  |  | N | 12 | Providing meals at foster facilities that house children whose parents died unexpectedly |
