# Supplementary material for: Altruistic responses to the most vulnerable involve sensorimotor processes
Source: Front Psychiatry. 2023 Mar 10;14:1140986. doi: 10.3389/fpsyt.2023.1140986 (PMC10036353; doi:10.3389/fpsyt.2023.1140986)
Supplement: Supplementary file 2 [file Table_2.docx]

**Table S2. Task > Baseline Whole Brain Effects.**

| **Period** | **Area** | **Voxels** | ***p*_FWE_** |  | **t** | **x** | **y** | **z** |
| --- | --- | --- | --- | --- | --- | --- | --- | --- |
| Charity | IFG/AI | 430 | < .001 | *** | 10.9 | 34 | 26 | -4 |
|  | Cerebellum/IOG | 3240 | < .001 | *** | 9.8 | -16 | -92 | -20 |
|  | Lingual gyrus | - | < .001 | *** | 9.3 | -6 | -98 | -10 |
|  | Lingual gyrus | - | < .001 | *** | 8.8 | 18 | -94 | -12 |
|  | dACC | 4000 | < .001 | *** | 8.9 | 10 | 24 | 40 |
|  | MFG/DMPFC | - | < .001 | *** | 8.8 | -4 | 14 | 46 |
|  | preCG/M1 | - | < .001 | *** | 8.8 | -40 | -4 | 58 |
|  | AI | 435 | < .001 | *** | 8.8 | -32 | 24 | -4 |
|  | MFG/PM | 1173 | < .001 | *** | 8.6 | 34 | -2 | 62 |
|  | MFG/PM | - | .002 | ** | 6.0 | 48 | 8 | 50 |
|  | postCG/S1 | - | .003 | ** | 5.8 | 44 | -26 | 48 |
|  | SPL | 940 | < .001 | *** | 8.1 | -30 | -58 | 46 |
|  | SPL | 685 | < .001 | *** | 7.8 | 34 | -54 | 50 |
|  | - | - | < .001 | *** | 6.6 | 26 | -64 | 50 |
|  | MFG/DLPFC | 565 | < .001 | *** | 7.1 | 52 | 30 | 30 |
|  | IFG | - | .001 | ** | 6.3 | 46 | 6 | 28 |
|  | - | - | .008 | ** | 5.5 | 60 | 10 | 30 |
|  | IPL | 146 | < .001 | *** | 6.7 | -44 | -34 | 42 |
|  | MTG | 90 | .002 | ** | 6.0 | -58 | -46 | 0 |
|  | SOG | 30 | .009 | ** | 5.4 | -30 | -78 | 24 |
|  | dStriatum / Caudate | 18 | .019 | * | 5.2 | 12 | 8 | 6 |
|  | Red nucleus | 10 | .026 | * | 5.1 | 4 | -28 | -6 |
| Donation | IPL | 32370 | < .001 | *** | 13.6 | 48 | -38 | 56 |
|  | Declive / V6 | - | < .001 | *** | 12.5 | -40 | -82 | -16 |
|  | Fusiform gyrus | - | < .001 | *** | 12.5 | 22 | -86 | -16 |
|  | MFG | 3536 | < .001 | *** | 9.9 | -44 | 46 | -10 |
|  | STG | - | < .001 | *** | 9.1 | -46 | 20 | -16 |
|  | IFG/VLPFC | - | < .001 | *** | 9.1 | -44 | 28 | -12 |
|  | Thalamus, posterior | 1213 | < .001 | *** | 9.0 | 24 | -28 | 0 |
|  | Ventral lateral thalamic nucleus | - | < .001 | *** | 7.0 | 16 | -10 | 6 |
|  | Mamillary bodies / Hypothalamus | - | < .001 | *** | 6.7 | 0 | -8 | -12 |
|  | ITG | 316 | < .001 | *** | 8.1 | -62 | -28 | -16 |
|  | - | - | < .001 | *** | 7.8 | -60 | -20 | -20 |
|  | - | - | .001 | ** | 6.2 | -60 | -42 | -14 |
|  | Thalamus, posterior | 181 | < .001 | *** | 7.3 | -24 | -28 | -2 |
|  | postCG/Precuneus | 297 | < .001 | *** | 6.4 | 2 | -28 | 32 |
|  | Rectal gyrus/OFC, anteromedial | 47 | .001 | ** | 6.3 | 2 | 36 | -20 |
|  | SFG/OFC, anterior | - | .004 | ** | 5.7 | -8 | 54 | -20 |
|  | dStriatum/Caudate | 18 | .018 | * | 5.2 | 14 | 10 | 12 |
|  | Substantia nigra, midbrain | 9 | .021 | * | 5.2 | -10 | -22 | -8 |
|  | Posterior pons | 4 | .028 | * | 5.1 | 6 | -34 | -34 |
|  | MFG | 2 | .032 | * | 5.0 | -12 | 60 | -14 |
|  | Cuneus | 2 | .045 | * | 4.9 | -28 | -92 | 22 |
|  | SFG/OFC, anteromedial | 1 | .050 | * | 4.9 | -8 | 58 | -18 |

Whole-brain results for Charity Viewing and Donation periods compared to the resting baseline, presented at p < .05 with a family-wise error (FWE) correction for multiple comparisons. ***p < .001, **p < .01, *p < .05. AI = anterior insula, CMA = cingulate motor area, dACC = dorsal anterior cingulate cortex, DLPFC = dorsal lateral prefrontal cortex, DMPFC = dorsal medial prefrontal gyrus, dStriatum = dorsal striatum, IFG = inferior frontal gyrus, IOG = inferior occipital gyrus, IPL = inferior parietal lobule, ITG = inferior temporal gyrus, M1 = primary motor area, MFG = middle frontal gyrus, MTG = middle temporal gyrus, OFC = orbitofrontal cortex, PM = premotor area, PMd = dorsal premotor area, postCG = postcentral gyrus, preCG = precentral gyrus, S1 = primary somatosensory cortex, SFG = superior frontal gyrus, SFGmed = medial superior frontal gyrus, SMA = supplementary motor area, SOG = superior occipital gyrus, SPL = superior parietal lobule, STG = superior temporal gyrus, VLPFC = ventral lateral prefrontal cortex.
