# Supplementary material for: Altruistic responses to the most vulnerable involve sensorimotor processes
Source: Front Psychiatry. 2023 Mar 10;14:1140986. doi: 10.3389/fpsyt.2023.1140986 (PMC10036353; doi:10.3389/fpsyt.2023.1140986)
Supplement: Supplementary file 3 [file Table_3.docx]

**Table S3. Main effects and interactions.**

| **Analysis** | **Period** | **Contrast** | **Gyral** | **Functional/Subarea** | **Voxels** | **t** | **x** | **y** | **z** |
| --- | --- | --- | --- | --- | --- | --- | --- | --- | --- |
| 2x2 Effect of Condition | Charity | Immediate Response > Preparatory Support | SFG/MFG | PMd* | 31 | 3.26 | -34 | 0 | 56 |
|  |  |  | SFG/MFG | PMd* | - | 3.19 | -36 | -4 | 54 |
|  |  |  | SFG/MFG | PMd* | - | 2.63 | -32 | -2 | 48 |
|  |  |  | SFG/MFG | preSMA | 23 | 3.05 | -8 | 16 | 52 |
|  |  |  | STG | STG | 837 | 4.78 | -54 | -50 | 14 |
|  |  |  | MTG | MTG | - | 4.42 | -50 | -52 | 6 |
|  |  |  | MTG | MTG | - | 4.39 | -48 | -48 | 4 |
|  |  | Nurturant > Heroic | STG | STG | 50 | 3.05 | -48 | -46 | 8 |
|  | Donation | Preparatory Support > Immediate Response | dStriatum | Caudate | 5 | 2.65 | -14 | 18 | -2 |
| Added regressor for Neonate vs. Adult | Donation | Immediate slope, Neonate > Adult | SFG/MFG | preSMA | 4 | 2.76 | -10 | 22 | 50 |
|  |  | Heroic slope, Neonate > Adult | vStriatum | vStriatum | 5 | 2.81 | 2 | 0 | -12 |

Additional analyses investigating continuous regressors for preparatory vs. Immediate, nuturant vs. heroic, and neonate vs. adult. All significant regions emerged from the *a priori* ROI from the prior literature (50) except for the cluster in PMd (indicated with an asterisk*), which was from the retrieval localizer ROI. dStriatum = dorsal striatum, MFG = middle frontal gyrus, PMd = dorsal premotor area, preSMA = area anterior to supplementary motor area, SFG = superior frontal gyrus, STG = superior temporal gyrus, MTG = middle temporal gyrus, vStriatum = ventral striatum.
